# Contribution of Increased Expression of Yin Yang 2 to Development of Cardiomyopathy

**DOI:** 10.3389/fmolb.2020.00035

**Published:** 2020-03-03

**Authors:** Yi Zhang, Ilimbek Beketaev, Ana Maria Segura, Wei Yu, Yutao Xi, Jiang Chang, Yanlin Ma, Jun Wang

**Affiliations:** ^1^The First Affiliated Hospital of Hainan Medical University, Haikou, China; ^2^Stem Cell Engineering, Texas Heart Institute, Houston, TX, United States; ^3^Department of Cardiac Pathology, Texas Heart Institute, Houston, TX, United States; ^4^Department of Biochemistry and Molecular Biology, University of Houston, Houston, TX, United States; ^5^Laboratory of Electrophysiology, Texas Heart Institute, Houston, TX, United States; ^6^Center for Translational Cancer Research, Institute of Biosciences and Technology, Texas A&M Health Science Center, Houston, TX, United States; ^7^Key Laboratory of Tropical Translational Medicine of Ministry of Education, Hainan Medical University, Haikou, China

**Keywords:** Yin Yang 2, gain of function, cardiomyopathy, autophagy, JNK

## Abstract

Yin Yang 2 (YY2) is a member of the Yin Yang family of transcription factors. Although the bioactivity of YY2 has been previously studied, its role in cardiovascular diseases is not known. We observed the increased expression of YY2 in failing human hearts compared with control hearts, raising the question of whether YY2 is involved in the pathogenesis of cardiomyopathy. To investigate the potential contribution of YY2 to the development of cardiomyopathy, we crossed two independent transgenic (Tg) mouse lines, pCAG-YY2-Tg+and alpha-myosin heavy chain-cre (α-MHC-Cre), to generate two independent double transgenic (dTg) mouse lines in which the conditional cardiomyocyte-specific expression of YY2 driven by the α-MHC promoter was mediated by Cre recombinase, starting at embryonic day 9.0. In dTg mice, we observed partial embryonic lethality and hearts with defective cardiomyocyte proliferation. Surviving dTg mice from both lines developed cardiomyopathy and heart failure that occurred with aging, showing different degrees of severity that were associated with the level of transgene expression. The development of cardiomyopathy was accompanied by increased levels of cardiac disease markers, apoptosis, and cardiac fibrosis. Our studies further revealed that the Cre-mediated cardiomyocyte-specific increase in YY2 expression led to increased levels of Beclin 1 and LC3II, indicating that YY2 is involved in mediating autophagic activity in mouse hearts *in vivo*. Also, compared with control hearts, dTg mouse hearts showed increased JNK activity. Because autophagy and JNK activity are important for maintaining cardiac homeostasis, the dysregulation of these signaling pathways may contribute to YY2-induced cardiomyopathy and heart failure *in vivo*.

## Introduction

The Yin Yang family member protein Yin Yang 2 (YY2) shares 56% amino acid sequence identity and 86% similarity in the zinc finger domain with YY1 (Nguyen et al., 2004). In contrast to the well-defined function of YY1 in DNA replication, cell cycle regulation, cell death, and organogenesis, little is known about YY2’s biologic function. In several studies, YY1 and YY2 have been shown to bind to a similar DNA sequence [i.e., A/CCAT (Yant et al., 1995)] and share multiple common targets, as revealed by the genome-wide analysis of human YY1 and YY2 shRNA knockdown cell lines (Kim et al., 2007; Chen et al., 2010). Others have shown that YY2 antagonizes YY1’s effects on the promoters of interleukin 4 *(IL-4*) and interferon beta (*INF-b*) genes (Klar and Bode, 2005; Lee et al., 2016), suggesting that YY1 and YY2 may have distinctly different activities. To date, most studies of YY2 activity have involved *in vitro* approaches. One study showed that the increased expression of YY2 impairs primary neuron differentiation and triggers cell death (Klar et al., 2015). More recently, the methylation of lysine 247 of YY2 (K247) has been shown to mediate cell proliferation and is potentially involved in cancer growth (Wu et al., 2017).

Previous studies have shown that YY1 plays an important role in maintaining adult cardiac function and in cardiac disease development (Gregoire et al., 2013; Beketaev et al., 2015), but whether YY2 has any role in cardiovascular disease remains unknown. Cardiomyopathy, a cardiac muscle disorder, is a leading cause of heart failure (Whelan et al., 2010). Dilated cardiomyopathy is one of the most common types of cardiomyopathy, characterized by the impaired capacity of the left ventricle to pump blood. Because dilated cardiomyopathy is a major health issue worldwide, uncovering the molecular basis of this disease to develop effective methods of treatment is an area of intense interest. Although the development of dilated cardiomyopathy is believed to involve multifactorial mechanisms including genetic factors and environmental cues (McNally and Mestroni, 2017; Weintraub et al., 2017), a tremendous amount of effort has been invested in elucidating the underlying mechanisms, including the signaling pathways and molecules, that have causative links to the pathogenesis and progression of dilated cardiomyopathy. YY1 is required for normal cardiogenesis, as evidenced by the cardiac structural defects observed in the embryonic hearts of mice with the cardiomyocyte-specific knockout of *YY1*(Gregoire et al., 2013; Beketaev et al., 2015).However, in failing human hearts, YY1 expression has been found to be increased and is believed to cause functional damage through the repression of alpha myosin heavy chain (α-MHC) promoter activity (Sucharov et al., 2004). On the other hand, the same group showed that YY1 prevented pathologic hypertrophy through HDAC5 (Sucharov et al., 2008). Interestingly, YY2 has been shown to be constitutively expressed in the embryonic and adult hearts of mice (Drews et al., 2009). Furthermore, the overexpression of YY2 in mouse embryonic stem cells has been shown to drive the expression of cardiovascular genes (Tahmasebi et al., 2016). However, whether YY2 has a role in cardiac disease is not known.

In this study, we investigated the role of YY2 in cardiomyopathy and its underlying molecular mechanisms by using a conditional gain-of-function mouse model in which we induced the cardiomyocyte-specific expression of YY2 with Cre recombinase under the control of the a-MHC promoter. Our findings show that the conditional cardiac expression of YY2 induces partial embryonic lethality and results in cardiomyopathy and heart failure that occurs with aging.

## Results

### Expression of YY2 Is Increased in Failing Human Hearts

**FIGURE 1:**
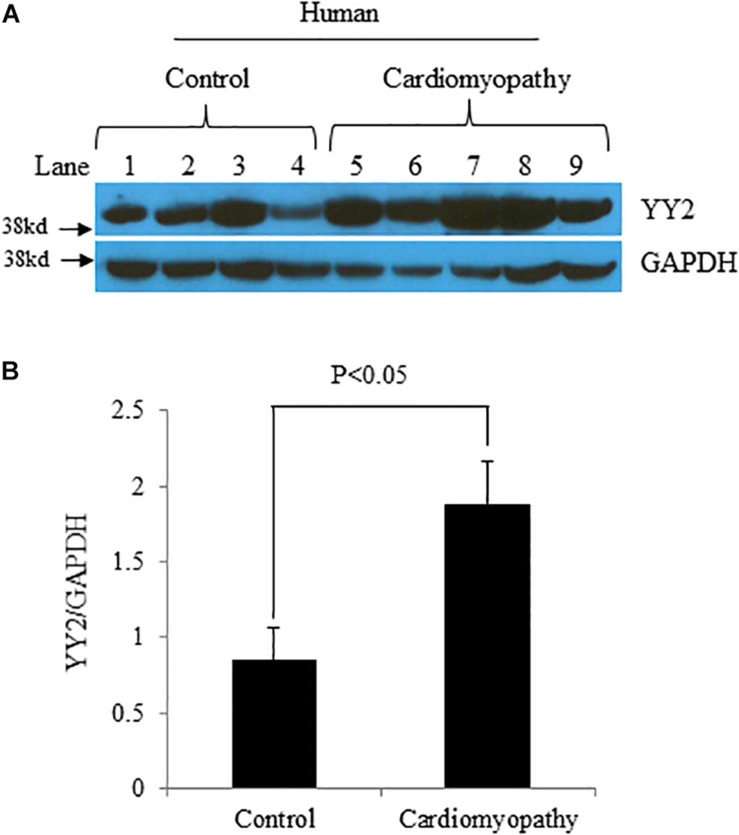
Increased levels of YY2 in failing human hearts.**(A)** Western blot analysis was performed with protein lysates that were collected from the left ventricle (LV) of either patients with heart failure caused by idiopathic cardiomyopathy or controls. GAPDH was used as an internal control. **(B)** Quantitative analysis of Western blot data shown in **(A)** (*n* = 4 for the control group and *n* = 5 for the cardiomyopathy group).

We first evaluated the expression levels of YY2 in control hearts (n = 4) and hearts with cardiomyopathy (n = 5) from patients with end-stage heart failure. These patients specifically had idiopathic cardiomyopathy, which was diagnosed on the basis of clinical symptoms and cardiac functional analysis. Western blot analysis showed that the expression levels of YY2 were significantly higher in hearts with cardiomyopathy than in control hearts (Figures 1A,B, *p* < 0.05).

### Increased Expression of YY2 in Cardiomyocytes Causes Partial Embryonic Death in Mice

**FIGURE 2:**
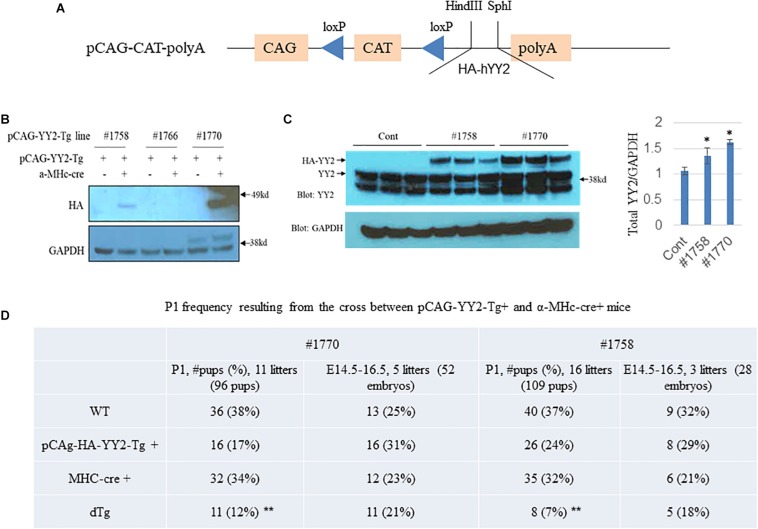
Generation of inducible YY2 transgenic mice. **(A)** A schematic diagram showing the inducible YY2 construct that contains HA-tagged human YY2 (HA-hYY2). **(B)** Two independent transgenic mouse lines, #1758 and #1770, were identified. Western blot analysis was performed with heart lysates as described in Figure 1. Upper panel: immunoblotting with HA-HRP; lower panel: immunoblotting with GAPDH-HRP (loading control). **(C)** Western blot analysis shows that the cardiac expression levels of the HA-YY2 transgene and endogenous YY2 in mouse line #1758 and line #1770 (*n* = 3 per group). ^∗^*p* < 0.05 vs. cont. **(D)** Partial embryonic death was observed in mouse embryos overexpressing YY2. Timed mating was performed between pCAG-YY2-Tg+and MHC-Cre+mice; the P1 frequency of double Tg (dTg; pCAG-YY2-Tg+/MHC-Cre+) mice from either of the pCAG-YY2 Tg mouse lines was significantly lower than the expected Mendelian rate. ^∗∗^ *p* < 0.01 vs. WT.

To explore the role of YY2 in the initiation and progression of cardiomyopathy, we generated an inducible gain-of-function mouse model in which the expression of HA-tagged YY2 (HA-YY2) was under the control of Cre recombinase (Figure 2A). In this model, Cre expression was driven by the a-MHC promoter in cardiomyocytes only, starting at ∼embryonic day (E) 9.0. Three independent pCAG-YY2-Tg mouse lines (i.e., #1758, #1766, and #1770) showed the transmittable transgene. When those mice were crossed with a-MHC-Cre mice to generate double transgenic (dTg) mice, only the mice generated from pCAG-YY2-Tgmouse lines #1758 and #1770 displayed the cardiac expression of HA-YY2 (Figure 2B). The expression level of HA-YY2 in the hearts of dTg mice generated from mouse line #1758 was lower than that in hearts of dTg mice generated from mouse line #1770 (Figure 2B). Compared with endogenous myocardial YY2 expression in control mice, the cardiac expression of YY2 (i.e.,endogenous YY2 + HA-YY2) was increased by ∼20% in mouse line #1758 and by ∼60% in mouse line #1770 (Figure 2C). Next, we examined the frequency of postnatal day (P) 1 (P1) resulting from the crossing of either inducible mouse line with the a-MHC-Cre mouse line. For pCAG-YY2-Tg mouse line #1770, we obtained 11 YY2-Tg+/a-MHC-Cre+dTg newborns out of a total of 96 pups (12%), a rate that was significantly lower than the expected 25% rate at P1. The other three genotypes, WT, pCAG-YY2-Tg+, and a-MHC-Cre+, were represented at rates that were comparable to or even higher than the expected 25% rate (Figure 2D). Similar results were obtained from crossing pCAG-YY2-Tg mouse line #1758 and the a-MHC-Cre+mouse line. These findings suggest that the expression of YY2 that was induced in murine cardiomyocytes at E9.0 caused partial embryonic lethality. To further determine the time of death of the dTg embryos, we collected and genotyped embryos between E14.5 and E16.5 that resulted from crossing either inducible Tg line with a-MHC-Cre mice. For embryos from either line, the ratio of dTg embryos to total embryos was close to the expected 1:4 ratio (25% rate), at 21% for line #1770 and 18% for line #1758. Thus, the embryonic death of dTg mice occurred after the embryonic stage E16.5.

### Induced Expression of YY2 in Murine Cardiomyocytes Impairs Cardiomyocyte Proliferation

**FIGURE 3:**
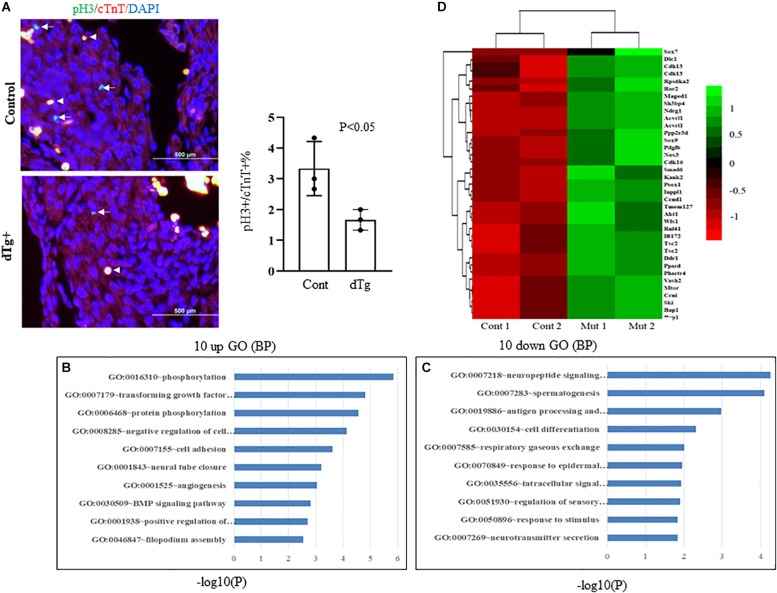
Impairedcell proliferation in double transgenic (dTg) mouse hearts.**(A)** Right panel: immunofluorescence staining was performed with sections from E14.5 control and dTg mouse embryos by using anti-pH3 (pH3: green, a cell proliferation marker) and anti-cTnT-AF647 (cTnT: red, a cardiomyocyte marker). DAPI (blue) was used for staining nuclei. Arrows indicate pH3+/cTnT+cardiomyocytes. Arrowheads indicateexamples of stained red blood cells. The left panel shows the statistical analysis of the data shown in the right panel of **(A)**. Cont, control. Four randomly selected fields from each sample were scored for pH3^+^/cTnT^+^ cardiomyocytes (*n* = 3 per group). **(B,C)** Microarray analysis was performed with RNA purified from E14.5 control and dTg mouse embryos. The 10 most upregulated **(B)** and 10 most downregulated GO terms **(C)** are shown. **(D)** A heat map showing the dysregulated genes involved in cell cycle progression.

To determine the molecular basis of the cardiac phenotypes observed in the dTg embryos, we first analyzed cell proliferation in control and dTg hearts by staining sections of E14.5 embryonic heart tissue with an antibody against serine 10-phosphorylated histone H3 (pH3), a widely used specific marker for cell proliferation. Immunofluorescence staining against cardiac troponin T (cTnT) was used as a specific marker for cardiomyocytes. As shown in Figure 3A, the number of pH3^+^/cTnT^+^ cells per 100 cells in the dTg mouse hearts was significantly lower than that in the control hearts (*p* < 0.05), suggesting that the induced expression of YY2 impaired cell proliferation in mouse cardiomyocytes. TUNEL staining revealed no significant difference in cardiomyocyte apoptosis between control and dTg hearts at E14.5 (data not shown). To systematically analyze the molecular mechanisms leading to the cardiac phenotypes observed in dTg embryos, we performed microarray analysis of RNA purified from E14.5 control and dTg mouse hearts, and the results were analyzed by using DAVID GO gene functional classification first^1^. The 10 most upregulated and 10 most downregulated GO terms are shown in Figures 3B,C, respectively. One interesting finding was that the genes associated with the fourth most upregulated GO term were labeled “negative regulation of cell proliferation,” which was consistent with the deficient proliferation observed in embryonic dTg mouse hearts. The genes related to the dysregulation of proliferation are shown in Figure 3D. Collectively, these observations suggest that YY2 mediates the cell cycle progression of cardiac cells during mouse embryogenesis.

**FIGURE 4:**
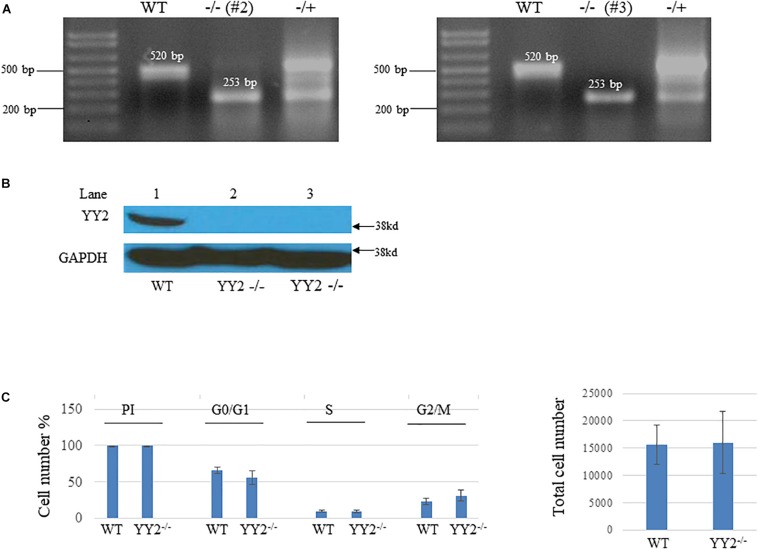
Generation of *YY2* knockout (KO) Hela cells by using the CRISPR/Cas9-mediated strategy.**(A)** PCR results showing the genotype of *YY2* KO Hela cells. The sizes of wild-type (WT) and *YY2* KO PCR bands are indicated. **(B)** Western blot analysis was performed to detect YY2 in cell lysates prepared from wild-type (WT) and *YY2* KO Hela cells. GAPDH was used as an internal control. **(C)** Results of flow cytometry analysis showing that *YY2* knockout did not dysregulate the cell cycle progression of Hela cells. *n* = 5 per group. PI, propidium iodide.

We then examined whether YY2 has a general role in cell cycle regulation. We generated CRISPR/Cas9-mediated YY2 knockout Hela cells by designing two guided RNAs (gRNAs) that guide Cas9 to delete a 267-bp fragment encoding YY2 between them (Figure 4A). Western blot analysis revealed that two clones, #2 and #3, showed the absence of YY2 (Figure 4B). However, performing cell progression analysis of WT and *YY2*-null cells with flow cytometry did not reveal any significant differences between them with respect to the percentage of cells that were undergoing G0/G1 and S and G2/M phases (Figure 4C), suggesting that YY2 in the cultured Hela cell line was not critically involved in mediating cell cycle progression.

### Induced Expression of YY2 in Cardiomyocytes Promotes Cardiomyopathy in Adult Mice

**FIGURE 5:**
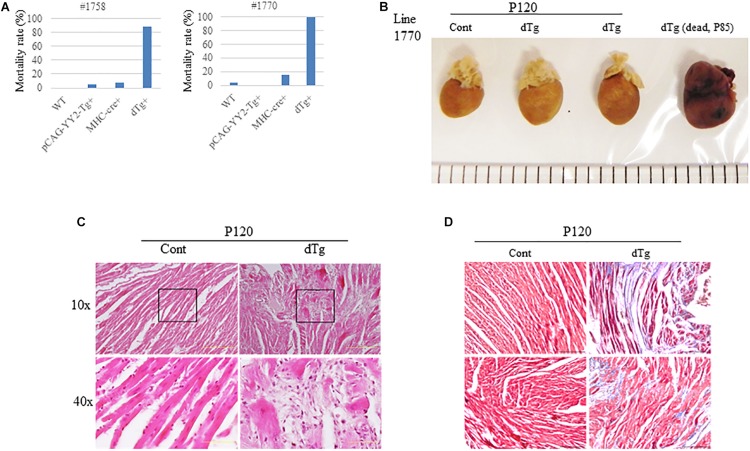
Adult cardiomyopathy resulting from the targeted activation of YY2 in mice.**(A)** The mortality rate in each genotype group within 1 year after birth is shown. The mortality rate was determined by calculating the number of deceased mice divided by the total number of mice analyzed in each group. For mouse line #1758 (*n* = 70), the following groups were analyzed: wild-type (WT) (*n* = 12), pCAG-YY2-Tg+(*n* = 20), MHC-cre+(*n* = 29), and dTg (*n* = 9). For mouse line #1770 (*n* = 87), the following groups were analyzed: WT (*n* = 31), pCAG-YY2-Tg+, (*n* = 23), MHC-cre+(*n* = 19), and dTg (*n* = 14). **(B)** Gross images of age-matched control and double transgenic (dTg) hearts from living mice euthanized at P120 and of a dTg heart from a mouse dead at P85. **(C)** Hematoxylin and eosin staining showed increased spaces between cardiomyocytes in dTg mouse hearts that were not observed in control hearts. The lower two panels represent magnified images of the respective boxed areas in the upper panels. Scale bar, 10 μm. Magnifications (10× and 40×) are indicated on the left side. **(D)** Masson’s trichrome staining showing increased fibrosis in dTg mouse hearts compared with control hearts. Heart sections were prepared from the mouse hearts shown in **(C)**. Two mouse hearts from each group are shown. Magnification, 40×; scale bar, 10 μm.

Although embryonic death occurred in some mice with cardiac-induced YY2 expression, a proportion of dTg mice from both lines survived to adulthood. However, within 1 year, all dTg mice from line #1770 and 88% of dTg mice from line #1758 died (Figure 5A). Morphologically, dTg hearts were slightly larger than the age-matched control hearts (Figure 5B, only line #1770 is shown), but the left ventricular (LV) mass/body weight ratio was not significantly different between groups (Tables 1,2). Cardiac functional analysis revealed that dTg mice from line #1770 and age-matched control mice had comparable cardiac functional parameters at ∼P43 such as ejection fraction (EF), fractional shortening (FS), and LV mass/body weight ratio (Table 1). However, at ∼P63, dTg mice from line #1770 developed impaired cardiac function, as evidenced by significantly lower EF and FS values than control mice, although no significant difference was observed in LV mass/body weight ratio between groups at this stage (Table 2). Compared with control mice, dTg mice from line #1758 had relatively normal cardiac function at ∼P135 but showed cardiac impairment at ∼P190 (Tables3, 4). These data suggested that dTg mice from line #1758 developed cardiac dysfunction at a later stage than did dTg mice from line #1770.

**TABLE 1:** Comparison of echocardiographic parameters between control and dTg mice at ∼P43 (mouse line #1770).

	Control^a^ (*n* = 7)	dTg^b^ (*n* = 4)	*P*-value
Age (days)	43.203.83	42.504.04	0.5269
IVSd (mm)	0.730.12	0.660.17	0.527
LVIDd (mm)	3.260.71	3.300.44	0.441
IVSs (mm)	1.050.22	0.990.27	0.985
LVIDs (mm)	2.190.79	2.400.57	0.655
LVPWd (mm)	1.010.19	0.960.16	0.680
LVPWs (mm)	1.270.23	1.170.20	0.474
Diameter; s (mm)	2.150.83	2.390.40	0.612
Diameter; d (mm)	3.370.71	3.490.27	0.760
Volume; s (μl)	20.339.52	20.678.39	0.955
Volume; d (μl)	41.9213.29	50.689.42	0.279
Stroke volume (μl)	30.308.13	30.025.65	0.952
Ejection fraction (%)	67.6815.17	59.9311.62	0.403
Fractional shortening (%)	37.8610.99	31.658.35	0.356
Cardiac output (ml/min)	11.453.54	10.963.26	0.825
LV mass (mg)	97.1934.59	87.1718.23	0.609
Body weight (g)	23.964.53	25.433.86	0.601
LV mass/body weight	4.021.06	3.420.38	0.308

*^*a*^Control group (two female and five male mice) included wild-type (*n* = 3); MHC-cre+(*n* = 2); and ^*b*^pCAG-YY2-Tg+(*n* = 2) mice.dTg group included two female and two male mice.*IVSd*,interventricular septum thickness at end-diastole; *IVSs*,interventricular septum thickness at end-systole; LVIDd, left ventricular internal dimension at end-diastole; *LVIDs*, left ventricular internal dimension at end-systole; LVPWd, left ventricular posterior wall thickness at end-diastole; LVPWs, left ventricular posterior wall thickness at end-systole; d, diastole; s, systole.*

**TABLE 2:** Comparison of echocardiographic parameters between control and dTg mice at ∼P63 (mouse line #1770).

	Control^a^ (*n* = 7)	dTg^b^ (*n* = 5)	*P*-value
Age (day)	63.291.89	64.61.95	0.268
IVSd (mm)	0.750.21	0.750.12	0.955
LVIDd (mm)	2.920.27	3.350.37	0.040*
IVSs (mm)	1.220.19	1.020.14	0.078
LVIDs (mm)	1.730.25	2.640.49	0.002**
LVPWd (mm)	0.980.12	1.190.37	0.187
LVPWs (mm)	1.400.21	1.320.20	0.529
Diameter; s (mm)	1.670.30	2.520.42	0.002
Diameter; d (mm)	2.950.27	3.370.46	0.076
Volume; s (μl)	8.393.69	23.6910.04	0.004**
Volume; d (μl)	34.057.26	47.5216.29	0.078
Stroke volume (μl)	25.655.06	23.839.68	0.677
Ejection fraction (%)	77.429.68	49.7613.95	0.002**
Fractional shortening (%)	43.876.73	25.088.62	0.002**
Cardiac output (ml/min)	11.462.36	9.323.97	0.267
LV mass (mg)	80.6523.20	117.4425.01	0.025*
Body weight (g)	22.331.94	21.841.80	0.668
LV mass/body weight	4.231.08	5.350.83	0.150

^*a*^Control group (four female and three male mice) included wild-type (*n* = 3), MHC-cre+(*n* = 1), and pCAG-YY2-Tg+(*n* = 3) mice. ^*b*^ dTg group contained three female and two male mice. *p < 0.05, **p < 0.01 vs. control.*IVSd*,interventricular septum thickness at end-diastole; IVSs, interventricular septum thickness at end-systole; LVIDd, left ventricular internal dimension at end-diastole; *LVIDs*, left ventricular internal dimension at end-systole; LVPWd, left ventricular posterior wall thickness at end-diastole; LVPWs, left ventricular posterior wall thickness at end-systole; d, diastole; s, systole.

**TABLE 3:** Comparison of echocardiographic parameters between control and dTg mice at ∼P135 (mouse line #1758).

	Control^a^ (*n* = 8)	dTg^b^ (*n* = 5)	*P*-value
Age (day)	142.2528.1	129.0028.17	0.426
IVSd (mm)	1.020.23	0.740.12	0.030*
LVIDd (mm)	3.220.67	3.040.20	0.595
IVSs (mm)	1.490.31	1.080.29	0.035*
LVIDs (mm)	1.920.60	1.910.33	0.968
LVPWd (mm)	1.340.49	1.450.34	0.668
LVPWs (mm)	1.750.52	1.890.33	0.623
Diameter; s (mm)	1.830.59	1.920.32	0.760
Diameter; d (mm)	3.270.64	3.120.25	0.645
Volume; s (μl)	11.929.64	12.054.48	0.978
Volume d (μl)	45.4319.90	38.927.19	0.502
Stroke volume (μl)	33.5213.90	26.873.75	0.325
Ejection fraction (%)	75.1913.47	69.847.47	0.438
Fractional shortening (%)	44.5111.97	38.776.35	0.349
Cardiac output (ml/min)	14.055.74	10.790.97	0.241
LV mass (mg)	144.8531.31	124.1839.64	0.317
Body weight (g)	32.304.50	31.53.73	0.747
LV mass/body weight	4.611.49	3.911.08	0.390

^*a*^Control group (two female and six male mice) included wild-type (*n* = 6); MHC-cre+(*n* = 1); pCAG-YY2-Tg+(*n* = 1). ^*b*^dTg group includes two female and three male mice. *p < 0.05 vs. control.*IVSd*,interventricular septum thickness at end-diastole; *IVSs*,interventricular septum thickness at end-systole; LVIDd, left ventricular internal dimension at end-diastole; *LVIDs*, left ventricular internal dimension at end-systole; LVPWd, left ventricular posterior wall thickness at end-diastole; LVPWs, left ventricular posterior wall thickness at end-systole; d, diastole; s, systole.

**TABLE 4:** Comparison of echocardiographic parameters between control and dTg mice at ∼P190 (mouse line #1758).

	Control^a^ (*n* = 6)	dTg^b^ (*n* = 4)	*P*-value
Age (day)	189.3317.6	194.5020.47	0.682
IVSd (mm)	0.850.14	0.600.11	0.016*
LVIDd (mm)	3.520.36	3.860.37	0.188
IVSs (mm)	1.260.18	0.810.23	0.009**
LVIDs (mm)	2.350.36	3.040.60	0.048
LVPWd (mm)	1.480.11	1.140.25	0.050
LVPWs (mm)	1.850.18	1.290.25	0.003**
Diameter; s (mm)	2.280.35	2.980.68	0.061
Diameter; d (mm)	3.650.46	3.960.47	0.327
Volume; s (μl)	18.317.10	36.6720.43	0.072
Volume; d (μl)	57.3516.75	69.6119.55	0.318
Stroke volume (μl)	39.0312.26	32.937.62	0.405
Ejection fraction (%)	68.077.37	499315.12	0.033*
Fractional shortening (%)	37.525.70	25.428.71	0.028*
Cardiac output (ml/min)	16.884.76	13.204.71	0.264
LV mass (mg)	164.2424.17	127.3944.81	0.126
Body weight (g)	38.725.22	34.807.73	0.362
LV mass/body weight	4.280.71	3.580.55	0.134

^*a*^Control group (two female and four male mice) included wild-type (*n* = 4) and pCAG-YY2-Tg+(*n* = 2) mice. ^*b*^dTg group includes two female and two male. *p < 0.05, **p < 0.01 vs. control.*IVSd*,interventricular septum thickness at end-diastole; *IVSs*,interventricular septum thickness at end-systole; LVIDd, left ventricular internal dimension at end-diastole; *LVIDs*, left ventricular internal dimension at end-systole; LVPWd, left ventricular posterior wall thickness at end-diastole; LVPWs, left ventricular posterior wall thickness at end-systole; d, diastole; s, systole.

**FIGURE 6:**
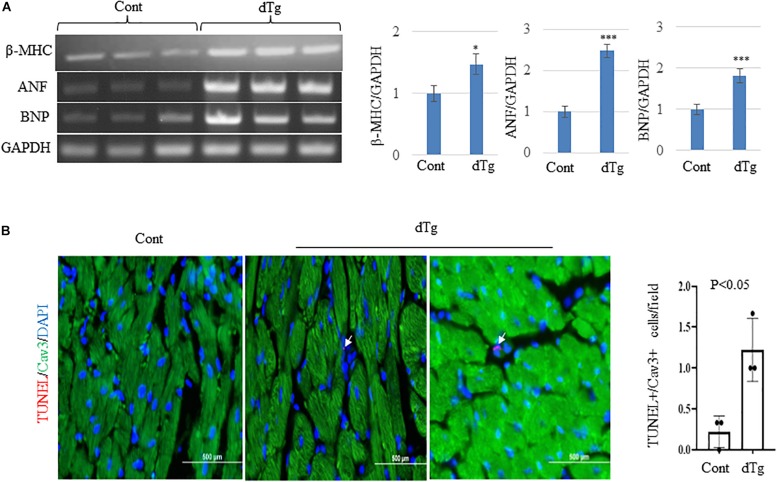
Increased expression of cardiac disease markers and apoptosis in double transgenic (dTg) mouse hearts.**(A)** Semi-quantitative PCR showing the change in expression of cardiac disease markers β-MHC, ANF, and BNP between control and dTg mouse hearts. The right panels show the quantitative analysis of the left, performed by using ImageJ. ^∗^*p* < 0.05, ^∗∗∗^*p* < 0.001 vs. Cont. Cont, control; dTg, pCAG-YY2-Tg+/α-MHC-Cre+.**(B)** TUNEL staining showing increased apoptosis in dTg hearts compared with control hearts at P50. TUNEL, red; caveolin3 (Cav3), green; DAPI, blue. Arrows indicate TUNEL+/Cav3+cardiomyocytes. *n* = 3 per group. ^∗^*p* < 0.05, vs. Cont.

**FIGURE 7:**
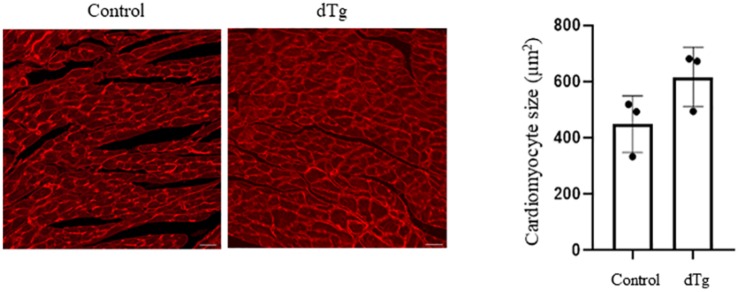
Cardiac hypertrophy not induced by the conditional expression of YY2. Wheat germ agglutinin (WGA)–tetramethylrhodamine (TRITC) staining was performed on heart sections prepared from control and dTg mice at ∼P65 to distinguish the sarcolemmal membrane. More than 100 randomly selected cardiomyocytes from each sample were used for surface area measurement using Software ImageJ. Magnification, 20×; scale bar, 10 μm. *n* = 3 per group.

We next performed the histopathologic analysis of heart sections prepared from age-matched control and dTg mice from line #1770. At P120, H&E staining revealed increased interspace between sarcomeres in dTg mouse hearts that were not observed in control hearts (Figure 5C), and Masson’s trichrome staining showed increased fibrosis in heart tissues from dTg+mice compared with control hearts (Figure 5D). These findings indicated that dTg mice developed cardiomyopathy that occurred with aging. In agreement with these findings, semi-quantitative PCR revealed the higher expression of cardiac disease markers β-myosin heavy chain (β-MHC), atrial natriuretic factor(ANF), and brain natriuretic peptide(BNP) in dTg mouse hearts than in control hearts at P50 (Figure 6A). Furthermore, TUNEL staining revealed the slight but significant increase in apoptosis in dTg mouse hearts compared with control hearts (Figure 6B). However, conditional expression of YY2 did not induce discernible cardiac hypertrophy in dTg mice (Figure 7). Thus, the induced expression of YY2 specifically in mouse cardiomyocytes promoted cardiomyopathy occurring with aging and premature demise.

### Mouse Hearts WithInduced YY2 Expression Show Increased Autophagic Activity

**FIGURE 8:**
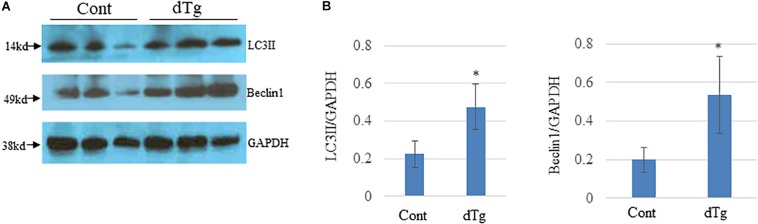
Increased autophagic flow in double transgenic (dTg) mouse hearts.**(A)** Western blot analysis was performed to detect LC3II and Beclin-1 in heart lysates from age-matched control and dTg mice. GAPDH was used as a loading control. **(B)** Quantitative analysis of data shown in **(A)**. ^∗^*p* < 0.05 vs. Cont, *n* = 3 per group.Cont, control; dTg, pCAG-YY2-Tg+/α-MHC-Cre+.

We next investigatedthe molecular mechanisms underlying YY2-induced cardiomyopathy. Because cardiomyopathy itself may cause changes in the activity of signaling pathways and molecules that are important for cardiac homeostasis, we analyzed heart samples of control and dTg mice from line #1770 at P50, when no significant cardiac phenotypes were observed. Autophagy is a highly conserved intracellular degradative process for protein recycling and has been shown to be involved in the pathogenesis of several human diseases, including heart diseases. In heart tissues from patients with dilated cardiomyopathy, increased autophagic activity has been observed and coincided with increased cell death (Shimomura et al., 2001; Kostin et al., 2003). Furthermore, in an animal model of heart failure, aortic banding was shown to induce cardiac hypertrophy that was accompanied by increased cardiac autophagy (Zhu et al., 2007). When we examined and compared autophagic activity in the hearts of age-matched control and dTg mice at P50, we found that levels of LC3II were significantly increased in dTg mouse hearts and coincided with the elevated expression of Beclin1, a critical regulator of autophagy (Figures 8A,B). These findings suggest that, in aggregates, YY2 expression increases autophagic activity in mouse hearts.

### Mitogen-Activated Protein Kinase (MAPK)SignalingIs Altered in dTg Mouse Hearts

**FIGURE 9:**
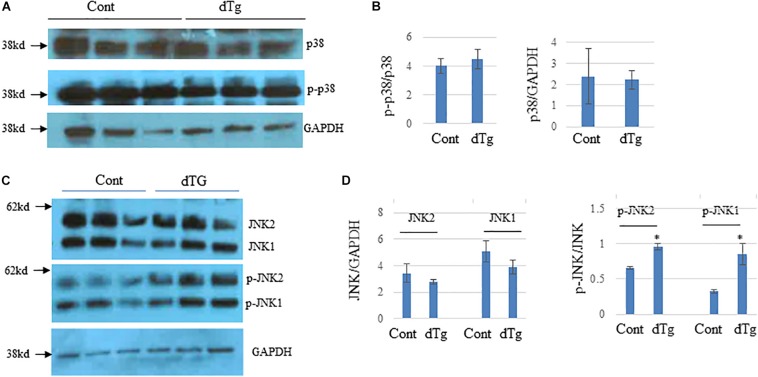
Altered JNK signaling pathways in double transgenic (dTg) mouse hearts. Western blot analysis was performed by using heart lysates from control and dTg mice. GAPDH was used as a loading control. **(A)** p38 activity was not significantly altered between age-matched control and dTg hearts. **(B)** Quantification of the data in **(A)**. **(C)** JNK activity was significantly higher in dTg hearts than in control hearts. **(D)** Quantification of the data shown in **(C)**. ^∗^*p* < 0.05 vs. Cont. *n* = 3 per group.Cont, control; dTg, pCAG-YY2-Tg+/-MHC-Cre+.

Because altered MAPK signaling has been implicated in the pathogenesis of several diseases including cardiomyopathy (Wang, 2007; Muslin, 2008), we examined MAPK signaling in the hearts of control and dTg mice. Western blot analysis showed no significant changes in the levels of p38, p-p38 (Figures 9A,B), or total JNK1/2 between groups (Figures 9C,D). However, dTg mouse hearts had significantly higher levels of p-pJNK1/2 than control hearts (Figures 9C,D), indicating increased MAPK signaling.

## Discussion

Previously, the first identified member of the Yin Yang family, YY1, was shown to have increased expression in failing human hearts (Sucharov et al., 2003) and to protect cultured cardiomyocytes from pathologic hypertrophy (Sucharov et al., 2008). However, whether YY2 plays a role in cardiovascular diseases had not been previously explored *in vivo*. To our knowledge, this is the first study to show that YY2 levels are elevated in failing human hearts and that activated YY2 promotes cardiomyopathy and heart failure in a conditional gain-of-function mouse model.

### YY2 and Cardiomyopathy

Although the role of YY1 in cardiomyocyte differentiation, cardiac development, and cardiomyopathy has been previously explored (Sucharov et al., 2003; Morikawa et al., 2013; Beketaev et al., 2015), studies of the involvement of YY2 in cardiovascular development and diseases have been limited, albeit that YY2 expression was detected in mouse hearts during both embryogenesis and postnatal stages (Drews et al., 2009). In a more recent study, it was revealed that the expression of YY2 induced by doxycycline promoted the differentiation of mouse embryonic stem cells into cardiovascular lineages (Tahmasebi et al., 2016), indicating an important role for YY2 in cardiac lineage commitment. In the present study, we found that the expression of YY2 induced by a-MHC-Cre, which was activated at ∼E9.0, caused partial prenatal lethality (i.e., after E16.5). The prenatal lethality of dTg mice was at least partially attributed to the deficient cell proliferation observed in the embryonic hearts of dTg mice. Our microarray analysis further confirmed that cell cycle genes were dysregulated in E14.5 dTg mouse hearts, as evidenced by the decreased pH3 staining in dTg hearts compared with control hearts. Given that YY2 depletion mediated by CRISPR/Cas9 caused peri-implantation lethality (Tahmasebi et al., 2016), we believe that YY2 plays a critical role in cell death and proliferation and that the level of YY2 should be tightly controlled during embryogenesis. Intriguingly, we did not observe any discernible cell cycle progression defect or increased apoptosis in CRISPR/Cas9-mediated *YY2* knockout Hela cells. Thus, knockout of YY2 in a cardiac cell line such as AC16 or specifically in cardiomyocytes *in vivo* is warranted to further explore the effect of loss of YY2 function on cardiomyocyte proliferation. We noted a recent study in which significant phenotypes were reported in *YY2*-null Hela cells that were also generated by using CRISPR-Cas9 technology (Wu et al., 2017). The reasons for this discrepancy were not clear but probably related to the different gRNA design and experimental protocols used.

In the present study, we found that the expression of YY2 was increased in failing human hearts. Furthermore, the ectopic cardiac-specific expression of YY2 recapitulated human cardiomyopathy in aging mice, showing different degrees of severity in two independent dTg mouse lines that correlated with the expression levels of the transgene. A previous study showed that the increased expression of YY2 in neurons led to increased cell death (Klar et al., 2015). Consistent with that report, we observed increased apoptosis before the development of cardiomyopathy in mouse hearts ectopically expressing YY2. Although multifactorial mechanisms underpin the pathogenesis of cardiac muscle disorders, increased apoptosis in cardiomyocytes, such as that seen here in dTg mouse hearts and in multiple other studies, may be a primary mechanism leading to cardiomyopathy (Narula et al., 2000; Whelan et al., 2010). Interestingly, both YY1 and YY2 are important for cell division and death. Given that knockout of either gene caused embryonic lethality, we believe that YY1 and YY2 have little overlapping function *in vivo*, at least with respect to embryonic development.

It should be noted that we used HA-tagged YY2 as a transgene to generate these two mouse lines in this study. It is a common practice to tag factors to generate genetically modified mice so that the transgene can be easily detected, as previously reported by our group and others (Ohnishi et al., 1997; Zou et al., 2001; Kim et al., 2011, 2014, 2015b). In another study from our group, HA-tagged YY2 significantly activated the target promoter (data not shown), indicating that HA-tagged YY2 maintains transcriptional activity. Given that the severity of cardiomyopathy in these two dTg mice was associated with the expression levels of HA-YY2, we believe that increased HA-YY2 was the cause of cardiomyopathy of these genetically modified mice in this study.

### YY2 and Autophagy

Heart samples from patients with dilated cardiomyopathy have been shown to have increased autophagy activity, which coincided with increased cell death (Shimomura et al., 2001; Hein et al., 2003; Kostin et al., 2003), whereas mechanical unloading in failing human hearts was shown to significantly decrease autophagic flux (Kassiotis et al., 2009). In addition, a lower occurrence of autophagic vacuoles—an indicator of autophagy—has been linked to myofilament changes in heart failure patients and is independently associated with death or readmission in these patients (Saito et al., 2016). Furthermore, in transgenic (Tg) mouse hearts that overexpress programmed cell death 5 (PDCD5) and have dilated cardiomyopathy (An et al., 2012), autophagy activity is increased. These findings indicate that the determinant of whether altered autophagic activity in the heart is beneficial or detrimental depends on the context. However, there is a general consensus that the tight control of autophagic activity is essential for the homeostasis of cardiomyocytes. In the present study, the increased cardiomyocyte-specific expression of YY2 caused cardiomyopathy, which was accompanied by increased autophagic activity, as evidenced by the increased expression of LC3II (an autophagic indicator) and Beclin1 (the major driver of autophagy). However, the role of YY2-induced autophagy in the pathogenesis of cardiomyopathy remains unclear; the haplo-insufficiency of Beclin1 (Beclin 1^±^), which is protective against cardiac remodeling in response to pressure overload in mice (Zhu et al., 2007), failed to ameliorate YY2-induced cardiac phenotypes (data not shown). Thus, the exact role that the YY2-induced increase in autophagy plays in the development of cardiomyopathy remains to be determined.

### YY2 and MAPK Signaling

The function of MAPKs, such as JNK and p38, in cardiac remodeling and disease development has been well described, although not without discrepancies. When JNK, a stress-activated protein kinase, is activated via the phosphorylation of its Thr-Pro-Tyr motif, it activates a large panel of downstream factors (Rose et al., 2010). Similar to JNK, once p38 is activated via the phosphorylation of its Thr-Gly-Tyr motif, it mediates a large spectrum of downstream cellular events such as apoptosis, proliferation, differentiation, and migration (Roux and Blenis, 2004). Previous studies have shown that the increased activation of JNK induces lethal cardiomyopathy and cardiac conduction defects (Petrich et al., 2002, 2004). Similar to JNK, the activation of p38 promotes cardiac remodeling and fibrosis and is associated with lamin A/C mutation-induced dilated cardiomyopathy (Liao et al., 2001; Muchir et al., 2012). In our mouse model of YY2 activation-induced cardiomyopathy, we did not observe any significant changes in protein expression of p38 or p-p38 between dTg and control hearts. In addition, we observed comparable levels of total JNK between control and dTg mouse hearts, although pJNK1 and pJNK2 levels were significantly increased in dTg mouse hearts. In consideration of previous findings, we speculate that increased JNK activity may be involved in the YY2-induced pathogenesis of cardiomyopathy. However, further studies involving JNK inhibitors in this mouse model are warranted.

In summary, we showed that YY2 levels are increased in failing human hearts and that the conditional cardiomyocyte-specific activation of YY2 in mice leads to partial embryonic lethality, which is associated with deficient cardiomyocyte proliferation and increased apoptosis, and aged-dependent cardiomyopathy, which is associated with altered activity of autophagy and MAPK signaling in the hearts. Thus, the cardiomyocyte-specific activation of YY2 in mice recapitulates human cardiomyopathy. Our findings indicate that YY2 is involved in promoting the pathogenesis of cardiomyopathy and suggest that YY2 may be a therapeutic target for the treatment of heart failure. Future studies will be needed to identify molecules that elicit age-dependent cardiomyocytes in the YY2 gain-of-function murine model.

## Materials and Methods

### Antibodies and Reagents

Antibody against horseradish peroxidase-conjugated HA (Anti-HA-HRP, Cat# A00169, 1:1000) was from Genscript (Piscataway, NJ, United States). Antibodies against GAPDH-HRP (Cat# sc-47724, 1:2000) and YY2 (Cat# sc-374455, 1:1000) were from Santa Cruz Biotechnology, Inc. (Dallas, TX, United States). The rabbit anti-YY2 antibody (Cat# A16621, 1:1000) was from ABclonal (Manhattan Beach, CA, United States). Anti-phospho-histone H3-Ser10 (pH3, Cat# ab10543, 1:200) was from Abcam (Cambridge, MA, United States). Alexa Fluor^®^ 647-conjugated mouse anti-cardiac troponin T (cTnT, Cat# 565744, 1:200) was from BD Sciences (San Jose, CA, United States). Antibodies against p38 (Cat# AF8691, 1:1000) and p38 phosphorylated on Thr180 and Tyr182 (p-p38, Cat# NB500-138, 1:1000) were from Novus Biologicals (Centennial, CO, United States). Antibodies against JNK (Cat# 9252, 1:1000) and JNK phosphorylated on Thr183 and Tyr185 (Cat# 46715, 1:1000) were from Cell Signaling Technology (Danvers, MA, United States). The fluorophore-conjugated secondary antibodies Alexa Fluor^®^ 488 (donkey anti-mouse, Cat# A-21202, 1:500) and 594 (goat anti-rabbit, Cat# A-11037, 1:500), lipofectamine 2000, lipofectamine 3000, and NuPAGE precast gels were from ThermoFisher Scientific (Carlsbad, CA, United States). ApopTag^®^ Red *In Situ* Apoptosis Detection Kit was purchased from MilliporeSigma (Burlington, MA, United States). T4 polynucleotide kinase, QuickLigase kit, DH5a, and NEBNext High-Fidelity 2xPCR Master Mix were from New England Biolab (Ipswich, MA, United States). HyGlo Quick Spray and Choice Taq Mastermix were from Denville Scientific, Inc. (Houston, TX, United States). ECL Plus was from GE Healthcare (Chicago, IL, United States). Immobilon^TM^ Western and polyvinylidene difluoride (PVDF) membrane were from MilliporeSigma. The Mini Prep Kit was from Qiagen (Hilden, Germany). Dulbecco’s Modified Eagle’s Medium (DMEM) was from Corning Inc. (Corning, NY, United States). Fetal bovine serum (FBS) was from Atlanta Biologicals (Flowery Branch, GA, United States).

### Mice

The Cre-inducible construct pISceI-CAG-CAT-polyA, in which CAT is flanked by two loxP sites, was a gift from Dr. Kimi Araki at Kumamoto University in Kumamoto, Japan (Araki et al., 1995). HA-tagged human YY2 was PCR-amplified and subcloned into pISceI-CAG-CAT-polyA vector via enzymatic digestion with *Eco*RV and *Sph*I (pCAG-HA-YY2). The insert was confirmed by sequencing, and YY2 expression was verified by performing co-transfection with pCAG-HA-YY2 and the pCMV-Cre expression vector and then performing Western blot analysis with an anti-HA antibody. The pCAG-HA-YY2 construct was then used to generate inducible Tg YY2 mouse lines (pCAG-YY2-Tg) by using the FBV mouse strain. The founders (F0) of pCAG-YY2-Tg mouse lines were backcrossed with C57BL/6 mice for one to five generations. The positive offspring were identified by using PCR with the following oligos: forward: 5′ CGGCACTCTTAGCAAACCTC 3′; reverse: 5′ CTAACCTCCCGTCTGCTGT 3′. The Tg mouse line specifically expressing Cre in the heart (a-MHC-Cre) was described previously (Beketaev et al., 2015). The wild-type (WT) and single Tg (i.e., either pCAG-YY2-Tg+or a-MHC-Cre+) were used as controls. Timed mating between a-MHC-Cre+and pCAG-YY2-Tg+mice was arranged as needed, and the day when a vaginal plug was seen was designated as embryonic day (E) 0.5. Embryos at various developmental stages such as E10.5, E11.5, E12.5, E16.5, or postnatal day (P) 1 were collected for genotyping to perform P1 frequency analysis. All experimental protocols involving mice were approved by the Institutional Animal Care and Use Committee (IACUC) of the Institute of Biosciences and Technology at Texas A&M Health Sciences Center.

### Human Samples

Heart samples from patients with idiopathic cardiomyopathy and from controls were obtained as previously described (Kim et al., 2015a, b). Briefly, diseased heart samples from patients with end-stage heart failure were supplied by the Department of Cardiac Pathology at the Texas Heart Institute, and the donated non-transplantable normal human heart samples (controls) were provided by the International Institute for the Advancement of Medicine on the basis of an official agreement with the Laboratory of Electrophysiology at the Texas Heart Institute. The protocol for the use of human heart samples was approved by the Institutional Review Board at CHI St. Luke’s Health—Baylor St. Luke’s Medical Center.

### Cell Culture and Transfection

Hela cells were cultured in DMEM supplemented with 10% FBS. Transient transfection was carried out on 6-cm plates with either lipofectamine 3000 [for single-cell sorting by flow cytometry (FACS Aria SORP, BD)] or lipofectamine 2000 (for Western blot analysis) according to the manufacturer’s instructions.

### Generation of YY2-Null Hela Cells

CRISPR/Cas9 technology was used to generate *YY2* knockout Hela cells. The following two pairs of complementary oligos containing 20-bp guide sequences were used: gRNA#1, forward: 5′-CACCGCCTCAGCGTTCTTTTTCCCA-3′; reverse 5′-AAACTGGGAAAAAGAACGCTGAGGC-3′; and gRNA#2, forward: 5′-CACCGACATAAGCATTTCCTGGTCG-3′; reverse 5′-AAACCGACCAGGAAATGCTTATGTC-3′. A distance of 267 bp between the two Cas9 cutting sites was designed for genome editing. Genotyping oligos (5′-TTCTCTGCAG CTCGCGCCTT-3′ and 5′-GGAGGGCCAACTGGTCCTC-3′) were designed to amplify a 520-bp length amplicon for the WT allele and a 253-bp amplicon for the mutant allele. The two pairs of complimentary oligos containing guide sequences were subcloned separately into pX458 by using previously described methods (Ran et al., 2013) with slight modification. Briefly, 100 μM of each oligo was phosphorylated by T4 polynucleotide kinase, annealed by boiling for 10 min, and then left at room temperature overnight. The annealed oligos were ligated into the *Bbs*I-cut pX458 (designated as pX458-YY2-gRNA#1 and pX458-YY2-gRNA#2, respectively) by using a QuickLigase Kit (NEB, United States). The ligation mixture was transformed into competent DH5a cells (NEB, United States). After purification with a Mini-Prep Kit (Qiagen), the desired clones were identified by sequencing. Hela cells were transfected with pX458-YY2-gRNA#1 and #2, and single clones were selected by using flow cytometry. The *YY2*^–/–^Hela cell clones were confirmed by using both PCR and Western blot analysis.

### Cell Cycle Studies

Cell cycle progression was analyzed by using flow cytometry with the propidium iodide (PI)/RNase staining method (Catalog No. 550825, BD Biosciences, San Jose, CA, United States) according to the manufacturer’s protocol. Briefly, WT and mutant cells (250,000 cells per group) were placed in one 6-cm plate on day 1. Cell cycle analysis with flow cytometry was performed on day 3.

### Microarray Assay, Reverse Transcription, and Semi-Quantitative PCR

RNA was extracted from embryonic and adult hearts of double Tg (i.e., dTg) mice and their littermate or age-matched controls by using Trizol according to the manufacturer’s instructions. Microarray service was provided by Phalanx Biotech (OneArray Express, San Diego, CA, United States). A reverse transcription reaction was performed by using 1 μg total RNA per reaction and cloned reverse transcriptase, followed by performing semi-quantitative PCR with gene-specific primers to detect β-MHC, ANF, BNP, and GAPDH, whose sequences were detailed previously (Zelarayan et al., 2008; Kim et al., 2012). The sequences of oligos used to detect the transcription of the transgene HA-YY2 were as follows: forward, 5′-ATGGCTTCTAGCTATCCTTATG-3′; reverse, 5′-CACCGTAGATCCAATTGCCATC-3′.

### Western Blot Analysis

Western blot analysis was performed as previously described (Kim et al., 2015b). Briefly, 40–80 μg of protein lysate was extracted from human or mouse left ventricles or from cultured cells. Protein lysates were boiled, separated by using 4–12% gradient NuPAGE gels, and transferred to PVDF membrane. The blots were incubated with the antibodies of interest. The protein bands were visualized with chemiluminescence by using HyGlo Quick Spray, ECL Plus, or Immobilon^TM^ Western, based on the intensity of signal obtained from the preliminary tests. The specific protein bands were quantified by using software ImageJ, and protein levels were normalized to those of GAPDH.

### Echocardiography

Mouse cardiac function was analyzed by using the two-dimensional M-mode of a Vevo 770 (Visual Sonics, Toronto, ON, Canada) as previously described (Kim et al., 2015b). Briefly, mice were anesthetized by inhalation of 1% isoflurane and rested on a warm pad. Chest hair was removed, and a probe was placed on the chest to record cardiac function indices. The investigator who performed cardiac function measurements and analyzed data was blinded to animal genotypes. The following cardiac functional parameters were measured: interventricular septum thickness at end-diastole; left ventricular internal dimension at end-diastole; interventricular septum thickness at end-systole; left ventricular internal dimension at end-systole;EF, FS, stroke volume, and cardiac output.

### Histopathology

Embryonic (i.e., E14.5) or adult mouse hearts were dissected, fixed overnight in 4% (for embryonic hearts) or 10% (for adult hearts) paraformaldehyde, dehydrated in increasing concentrations of ethanol (up to 100%), paraffin-embedded, and sectioned at 5-μm thickness. Heart sections were stained with hematoxylin and eosin (H&E) and Masson’s trichrome staining by using standard protocols.

### Immunohistochemistry

Immunostaining was performed with sections of embryonic and adult mouse hearts as previously described (Kim et al., 2015b). Briefly, tissue sections were deparaffinized, and antigen retrieval was performed in boiling sodium citrate buffer (pH = 6.0) for 10 min. The tissue sections were then incubated with primary antibody against pH3 (a cell proliferation marker) at 4°C overnight, followed by an incubation with secondary antibody (Alexa Fluoro^®^ 48) and AF647-conjugated cTnT antibody for 1 h at room temperature. Tissue sections were mounted by using Vectashield with 4′,6-diamidino-2-phenylindole (DAPI; Vector Laboratory, Burlingame, CA, United States). Terminal deoxynucleotidyl transferase dUTP nick end labeling (TUNEL) staining was performed by using the ApopTagH Red *In Situ* Apoptosis Detection Kit (Millipore) according to the manufacturer’s protocol, and Cav3 staining was used to indicate cardiomyocytes in TUNEL-stained images. The positive-stained cells were scored from at least three randomly selected fields.

### Cardiomyocyte Size Measurement

Wheat germ agglutinin (WGA)–tetramethylrhodamine (TRITC) staining was performed on heart sections prepared from control and dTg mice at ∼P65 to distinguish the sarcolemmal membrane. More than 100 randomly selected cardiomyocytes from each sample were used for the surface area measurement by using Software ImageJ.

### Statistical Analysis

Numerical data are presented as the mean ± standard error of the mean, and categorical data are presented as n and percentage. The unpaired Student’s *t*-test, Fisher’s exact probability test or Chi-square test was used to determine statistical significance between groups as applicable. A *p-*value (*p*) < 0.05 was considered as statistically significant and *p* < 0.01 as highly significant.

## Data Availability Statement

Microarray data has been deposited to the GEO – GSE145808.

## Ethics Statement

The studies involving human participants were reviewed and approved by IRB, St. Luke’s Episcopal Hospital. The animal study was reviewed and approved by IACUC, Texas A&M Health Science Center.

## Author Contributions

JW and YM: conceptualization. YZ, IB, and WY: methodology. YZ, IB, and JW: formal analysis. YZ, IB, WY, and JW: investigation. AS, YX, and JC: resources. JW: writing – original draft preparation, supervision, project administration, and funding acquisition.

## Conflict of Interest

The authors declare that the research was conducted in the absence of any commercial or financial relationships that could be construed as a potential conflict of interest.

## Footnotes

1 https://david.ncifcrf.gov/
